# Men in Papua New Guinea Accurately Report Their Circumcision Status

**DOI:** 10.1371/journal.pone.0123429

**Published:** 2015-04-13

**Authors:** Parana Hewage Mangalasiri Jayathunge, William John Hannan McBride, David MacLaren, Kelwyn Browne

**Affiliations:** 1 Cairns Clinical School, College of Medicine and Dentistry, James Cook University, Cairns, 4870, QLD, Australia; 2 College of Medicine and Dentistry, Cairns Campus, James Cook University, Cairns, 4870, QLD, Australia; 3 Rural Primary Health Services Delivery Project, National Department of Health, Port Moresby, Papua New Guinea; Rega Institute for Medical Research, BELGIUM

## Abstract

**Background:**

Male circumcision (MC) is a well-established component of HIV prevention in countries with high HIV prevalence and heterosexually driven epidemics. Delivery and monitoring of MC programs are reliant on good quality MC data. Such data are often generated through self-reported MC status surveys. This study examined self-reported MC status in comparison with genital photographs from men in Papua New Guinea (PNG).

**Methods:**

This retrospective non-interventional study collated self-reported MC status data from the ‘acceptability and feasibility of MC’ study at 4 sites in PNG during 2010–2011. Participants reported their MC status based on an 8-category photographic classification covering the range of foreskin cutting practices in PNG. Genital photographs of 222 participants from this study were independently classified by 2 investigators. The 8-category photographic classification was simplified into a 3 category classification of ‘no cut’, ‘straight cut’ and ‘round cut’ before comparing for agreement between self-reporting and investigator assessment using Cohen’s Kappa measure.

**Results:**

Using the 3-category classification, there was 90.6% (201/222) agreement between self-assessment and investigator classification (κ value 0.805). Of the discordant 9.4% (21/222), 3.6% (8/222) self-classified as having a cut foreskin (5 straight cut; 3 round cut) while investigators classified as having no cut; 4.1% (9/222) self-classified as having no cut while investigators classified them as having had a cut (6 straight cut; 3 round cut) and 1.8% (4/222) self-classified as having a round cut while investigators classified as having a straight cut. Given the great variety of foreskin cutting practices and appearances, feasible explanations are suggested for two-thirds (13/21) of these discordant results.

**Conclusions:**

This study demonstrates a high level of agreement between self-reporting and investigator assessment of MC status in PNG and suggests self-reporting of MC status to be highly reliable among men in PNG.

## Introduction

The HIV epidemic in Papua New Guinea (PNG) is the largest in the Oceania region. In 2012 the national adult HIV prevalence in PNG (age 15+) was estimated at 0.5% (0.4–0.7%) with 22,000 (18000–28000) people living with HIV[1]. The HIV epidemic in PNG is primarily linked to heterosexual transmission but exhibits substantial heterogeneity across the country[2]. Innovative strategies for HIV prevention, treatment and care are needed to address this complex public health issue in PNG where extreme geographical, linguistic and cultural diversity is present[3]. Male Circumcision (MC) was identified as a prioritized research area in prevention of HIV in the 2008–2013 PNG National AIDS Council Research Agenda[3].

MC is defined as the surgical removal of all or part of the prepuce (foreskin) covering the glans penis[4]. In PNG, multiple forms of circumcision and foreskin cutting have been described. Some forms involve circumferential cuts that result in the complete removal of the foreskin, others involve longitudinal cuts where the foreskin is cut but not removed [5–9]. The most common foreskin cut in PNG is the longitudinal cut, which most often involves a single dorsal slit without removing the prepuce, but exposing the inner aspect of the foreskin and the glans[10]. In some places in PNG longitudinal cuts are described as a “v” cut because of the resultant appearance of the modified foreskin[11]. Moreover, there are also non-traditional types of penile modification such as penile inserts (ball bearings, beads, plastics etc.) into the skin of the penile shaft [8, 9, 12, 13]. A multi-site study conducted in PNG in 2010 reported 47% of men had some form of longitudinal foreskin incision and 10% had full circumcision[14]. The 2006 PNG National HIV/AIDS Behavioural Surveillance Survey (BSS) reported some form of foreskin cutting or medical circumcision amongst 26% of truck drivers, 45% of farm-factory workers, 67% of military personnel, and 70% of port workers[12]. A BSS conducted in rural development enclaves in PNG between 2008–2010 reported that more than a third of the men (34.3%) had longitudinal cuts of their foreskin [15].

Voluntary Medical Male Circumcision (VMMC) is a component of comprehensive HIV prevention services in some countries with high HIV prevalence following recommendations of the World Health Organisation (WHO) in 2007 [16]. Among the priority countries identified by WHO, Lesotho and Malawi were initially hesitant to scale up VMMC procedures as their own national survey results seemingly demonstrated higher HIV prevalence among those who reported being circumcised [16]. MC data were mainly based on studies where men self-reported MC status. Both countries performed relatively few VMMC procedures during 2008–2012 (2.8% and 1.7% of WHO targets respectively), however this is now increasing [17, 18].

MC status in previous studies has been recorded by either (i) self-reporting of MC status (ii) genital examination by an expert/clinician, or (iii) a combination of both. Several studies that used a combination of self-report and genital examination in different settings have shown large discrepancies in results between these two methods [16]. Self-report was shown as a valid measure of MC status in homosexual men in Australia [19]. Data from 5 population-based studies in north-western Tanzania showed accuracy (p<0.001) in self-reported data although the authors described some tendency for MC to be over reported among those populations [20]. Lagarde and his colleagues observed that 14% of self-reported MC status were discordant from clinical examination in their community-based cross sectional study conducted in Westonaria district, South Africa[21]. A study conducted on adolescent boys in Houston, Texas in 2002 demonstrated that self-reported MC status was discordant by 31% and 35% among circumcised and uncircumcised groups respectively [22]. Authors highlighted that self-reported circumcision status was questionable because almost a quarter (27%) of their participants did not know their circumcision status [22].

A study conducted during Lesotho Defence Force applicant screening in 2009 used self-administered MC questionnaire followed by a physician-performed genital examination [16]. This study documented 27% of males self-reported as being circumcised. Of these, physical examination showed that only half (50%) were fully circumcised, about a quarter (27%) had a partial circumcision and the remaining quarter (23%) were not circumcised. According to the authors, their questionnaire did not allow participants to report `partial’ circumcision status “other than yes/no categorisation” to MC status which may have confused participants in reporting their circumcision status. The authors recommended adding partial MC categories along with graphics depicting forms of MC to improve the quality of the study [16]. Studies conducted in Zambia and Swaziland in 2009 demonstrated improved self-reporting of MC status among illiterate participants when they were provided with an illustration of circumcised and uncircumcised status (misreporting reduced from 13% without an illustration to 10% with an illustration) [23]. Similarly, Scholossberger et al in their publication in 1992 on “Early adolescent knowledge and attitudes about circumcision” reported an improved accuracy of self-report from 68% to 92% using visual aids to report circumcision status among adolescents in the United States [24].

The study presented in this paper was conducted with the objective of assessing the reliability of self-reporting of MC status of men in PNG. Reliability of self-reported MC data are vital in planning and delivering health services and HIV prevention programs for the general public in PNG and other populations.

## Methods

This study was carried out as a sub study of large multi-site study titled “Is male circumcision an acceptable and feasible intervention to reduce HIV transmission in Papua New Guinea?” conducted in 2010 in four sites of PNG; Pacific Adventist University (National Capital District), Porgera Joint Venture (Enga Province), Divine Word University (Madang Province) and Higaturu Oil Palms (Oro Province) [14, 25]. These sites represent places of work or study for men from all over PNG. All the participants of the study were aged 18 or older and provided their informed written consent before participating in the study. The research was conducted in compliance with human research ethics approvals from the PNG National AIDS Council, PNG (approval No RES10 0011) and James Cook University, Australia (approval No H3757).

Two 2 methods used to assess MC status in the study:

1. Questionnaire: Participants were offered a self-administered questionnaire that recorded data on demographics (age, education, province of origin, religious affiliation and employment), foreskin cutting status and related information such as method of foreskin cutting, logistics of foreskin cutting (setting and provider) and the beliefs associated with those practices. Participants, some with limited literacy had a trained interviewer administer the questionnaire, some others who were fully literate requested to have an interviewer read them questions from the questionnaire and then they verbally responded to each question. The question to record MC status used a series of seven actual photographs of the most common foreskin cutting practices in PNG. The participant was requested to mark the most relevant photograph ‘that most looks like your own foreskin’. There was an eighth option for ‘other’ with a request to draw a picture. The full questionnaire, including the series of 7 photographs can be accessed through http://www.biomedcentral.com/content/supplementary/1471-2458-13-818-s1.pdf.
2. Clinical examination and photograph: Following the questionnaire, participants were invited for an optional clinical examination by a health professional. During these optional clinical examinations, participants were asked if the clinician could record their circumcision status using digital photography. The photography was optional and not a condition of clinical examination.

At the two university sites, study participants were residents (students and staff) on the university campus. Participants completed the questionnaire in their homes or dormitories and returned completed questionnaires (in sealed envelopes). The invitation for optional clinical examination involved an extra step to travel to a nearby clinic or hospital at designated times.

At the two rural sites study participants were residents from the area who were visiting the local health centre (or a family member of someone visiting the health centre). The invitation for the optional clinical examination involved an immediate examination in the health centre—where photography was requested, but not a condition of clinical examination. Clinical advice, treatment and/or referral were offered for any condition the participant presented with, not just sexual health conditions. Through this process 266 (31%) of the 861 participants self-selected for the photographic component of the study.

Prior to analysis, the photographs were assessed for clarity and the poor quality photos (n = 32) were discarded from analysis. Similarly, those photographs that could not be matched with the participants due to unclear numbers (n = 5) were also removed from analysis. Accordingly, genital photographs from 229 participants from 4 sites were included in the analysis.

Photographs were analysed separately by two investigators with clinical backgrounds. They categorised the MC status of the photographs into 8 categories as shown in the questionnaire (The investigators were blinded to the participants’ own classifications during the process). After photographs were independently classified, the 2 investigators discussed the differences in photo classifications between them and reached a consensus. To avoid complicated comparison across closely related categories, the investigators summarised the 8 categories into 3 major categories as “no cut” (Category 1), “straight cut” (Category 2–6 and 8) and “round cut” (Category 7) before analysing the agreement level between participants and investigators.

All the data were coded and initially entered into MS Excel and transferred to SPSS v.20 statistical software before analysis. Agreement between participants’ reporting and investigators’ classifications of MC status was analysed using Cohen’s kappa statistical method. The confounding factors for the accord and discord were analysed using binary logistic regression.

## Results

Demographic characteristics of men in the study whose photographs were analysed are presented in Table 1. The median age of the men was 27 years (IQ range 22–33). The majority of photographs (93.5%) were from participants at the two rural sites (Porgera and Popondetta) with 64% from Popondetta. Almost 98% of participants identified as Christian and 54.3% were married. Primary school was the highest education for 58.6% of participants and 24.9% had attended secondary or high school. Manual/agricultural workers made up 67.6% of participants and 24.3% were employed in trade or technical work (Table 1).

**Table 1:** Demographic characteristic of participants.

Demographic characteristic		Study group	General population
Age in years (median)		27(IQ range 22–33)	NA
Site	DWU	6(2.6%)	NA
	PAU	9(3.9%)	NA
	Porgera	67(29.3%)	NA
	Popondetta	147(64.2%)	NA
Marital status	Single	96(41.9%)	NA
	Married living together	125(54.6%)	NA
	Married not in union	8(3.5%)	NA
Education	Non/elementary/primary	133(58.6%)	64%
	Secondary/high	57(24.9%)	12%
	Vocational/tech college	26(11.4%)	NA
	University	11(4.8%)	<1%
Employment	Unemployed	2.3%	1.8%
	Agriculture	67.6%	72.7%
	Industry	24.3%	3.6%
	Services	4.6%	22.7%

(NA-Not Applicable)

Of the 229 participants with both questionnaire data and genital photos, 19 (8%) reported that they had inserts or attachments and 39 (17%) had injected some kind of substance into the penis. Seven [7] were excluded from the analysis because the photos showed such grossly dysmorphic features (after being injected with oils or potions) that it was too difficult to classify the foreskin cutting status. Table 2 displays data from participants and the investigators on 3-category foreskin cutting classification.

**Table 2:** MC status classification and respective reporting of participants and investigators.

MC status	Participant N (%)	Investigators’ consensus N (%)
No foreskin cut	83(37.4)	82(36.5)
Straight cut	129(58.1)	134(60.8)
Round cut	10(4.5)	6(2.7)
Total	222(100)	222(100)

The Table 3 displays participants’ self-assessment and investigators’ assessment of photos using the 3 category classification. Agreement on MC status between participants and investigators (consensus) was present in 90.6% of all cases (kappa 0.805) (Table 3).

**Table 3:** Comparison of participants’ self-assessment of MC status against investigators’ (consensus) assessment of photographs.

		Investigators		
		No cut	Straight cut	Round cut
Participants	No cut	74(33.3%)	6(2.8%)	3(1.4%)
	Straight cut	5(2.2%)	124(55.9%)	0(0%)
	Round cut	3(1.4%)	4(1.8%)	3(1.4%)

Of the 222 participants in the final analysis 21 (9.4%) had a difference between self-classification and investigator (consensus) classification: 8/222 (3.6%) self-classified as having a cut foreskin (5 straight cut; 3 round cut) while investigators classified as having no cut; 9/222 (4.1%) self-classified as having no cut while investigators classified them as having had a cut (6 straight cut; 3 round cut); 4/222 (1.8%) self-classified as having a round cut while investigators classified them as having a straight cut (Table 3).

A regression analysis of socio-economic status/employment, education level and the potential confounding factor of age of the participant revealed that there is no significant association between these factors and accord/discord of foreskin cutting status (S1 Table).

## Discussion

This study provides the first evidence on the level of agreement for MC status between self-assessment and clinical assessment using photographs in PNG. The results demonstrate a high degree of agreement using a 3 category classification and suggests that self-assessment of MC status by men in PNG is highly reliable. High reliability in self-reported MC status has important implications for planning VMMC programmes as data can be used to accurately estimate the volume of surgical intervention and resources required and identify the individuals for whom the intervention is indicated.

On further investigation we suggest several important factors that may explain the different responses by participants and investigators for two–thirds 13/21 of discordant results (comprising 6.2% of overall participants). Disagreements may be because:

1. investigators classified photos as ‘round cut’ but participants reported ‘no cut’ in instances where participants had naturally short foreskins or wore the foreskin retracted behind the glans penis (n = 3)
2. investigators classified photos as ‘no cut’ but participants reported ‘straight cut’ in instances where there was such small cuts that it caused minimal change in the morphology of the foreskin (n = 5)
3. investigators classified photos as ‘straight cut’ but participants reported ‘round cut’ in instances where remnant foreskin was obvious in the photo but the glans may have resembled the ‘round cut’ photo in the questionnaire (n = 4)
4. investigators classified a photo as ‘straight cut’ but the participant reported ‘no cut’ in an instance where the participant may have had his foreskin retracted (n = 1)

For the remaining 8/222 of discordant classifications (comprising 3% of overall participants), we can offer no explanation. In these discordant cases: 5 participants self-reported “no cut” when there was clear evidence from the photo of a scar and/or foreskin remnant behind the glans; 3 participants self-reported “total removal of foreskin” when there was clear evidence from photo that the glans penis was totally covered with foreskin.

Therefore, overall this study has demonstrated direct agreement in 91% of cases and a plausible explanation for a further 6%. This leaves only 3% discordance between self-assessment and clinical assessment of photographs. This result showing 97% agreement between self-assessment and investigator assessment is in direct contrast to studies conducted in Africa where agreement can be as low as 50%[16]. The authors believe that the high level of agreement between self-report and investigator recordings in this study could be due to the higher awareness among PNG men of MC and foreskin cutting practices. It is possible that men in PNG discuss MC and foreskin cutting as these practices have a long cultural tradition in some regions and also may be due to the awareness programmes on HIV prevention by various health programmes. However, further research is needed to support these hypotheses.

We also analysed the level of agreement of self-report with individual investigators’ classifications across the eight different types of foreskin cuts. The level of agreement with anything other than a three category classification (no cut; straight cut; round cut) remained low (data not displayed). We believe that this could be explained by the complexity of the eight level classification system where each category contained only subtle differences from others. Classification levels 2–6 are all sub-variations of the longitudinal ‘straight cut’. For a decision to be made on which classification a participant belongs to, requires a subjective decision based on appearance and interpretation of the participant’s own penis and the photographs in the questionnaire. When classification levels 2–6 were grouped into the broader category of ‘straight cut’, the level of agreement between self-assessment and investigator assessment dramatically improved indicating a 3 level system to be more practical method to assess MC in this population.

We made some important observations on MC status reporting during the study. At the time of the initial photograph classification by investigators, there were variable assessments of photos from men who permanently wore the foreskin retracted behind the glans penis, which is not uncommon in some parts of PNG. In such cases, final classification was agreed to by consensus between investigators. We also noted that the participants reported slightly more ‘no cut” and “round cut” than investigators. Social desirability bias could be considered as one probable explanation for this discrepancy, although with such small numbers this may be due to random errors and/or variable literacy levels. Further, there is great diversity of social, cultural and spiritual practices across PNG’s 800 distinctly different language groups. Celebrating conformity within a group, but highlighting difference across these groups is an enduring feature of life in PNG. Therefore this may explain a context in where men are confident to report their own MC status, even if it is different than other men who participated in the study (who may be resident at the study site, but have originated from any one of the other 800 language groups).

MC practices and other associated sexual practices are informed by local cultural practices with MC/foreskin cutting a historically important initiation process to adulthood in some cultural groups [9]. Local culture also plays a role in deciding the type of foreskin cutting, as different cultural groups practice different forms of foreskin cutting modes [26]. Apart from foreskin “cutting”, injections and inserts into the foreskin and penile shaft were also common amongst this population. In such cases the penis appeared to be scarred and hardened and sometimes forming a sclerosing lipogranuloma, a fibrous tissue development due to mineral oil injection [26]. Some participants reported both a longitudinal foreskin cut and injecting substances into the remnant foreskin. Photographs from such participants were so difficult to classify because of their dysmorphic appearance that they were excluded from this study. We have no reason that these exclusions affected the overall results of this study.

Because of the great diversity of social, cultural and spiritual practices, with associated MC and sexual practices, the results of this study cannot be generalized across the entire PNG population. Furthermore, given only 31% of participants self-selected for the photographic component of the study, this also precludes generalisation across the whole population. However this study does provide strong evidence that there is a high level of agreement between self-report and clinician assessment of photographs in PNG. It further demonstrates that this method is feasible in PNG—however that anything beyond a 3 level MC category may prove to be impractical to assess levels of agreement. The results of this study emphasize that the accuracy of self-reporting of MC status could vary across different countries and different populations and the need for location specific studies that reflect culturally specific practices.

One of the novelties of this study was the analysis of photographs of participants for measuring agreement between self-report and investigator classification. An advantage was that the investigator could analyse the photograph at any time and seek a second opinion from another investigator to come to a consensus. A disadvantage is the photograph is just a two dimensional image of the penis and the investigator is unable to perform a clinical examination on the actual participant to assess MC status. This study was also able to use real photographs of the most common foreskin cutting practices in PNG in the questionnaire instead of descriptions using words, sketches or diagrams.

Having a large number of photographs for the analysis was a strength in this study. This provided freedom for investigators to exclude unclear photos and yet have sufficient numbers for statistical analysis. Several pictures taken of a penis from different angles provided additional information and further facilitated assessment of MC status. A limitation of this study was the recruitment of participants only from 4 sites and the results are not generalizable, although the sites were chosen because of migration from across PNG, which serves to improve the applicability and transferability of results.

As nations with high or moderate HIV prevalence act on WHO recommendations for VMMC programs, the need for reliable MC prevalence data have become more critical [16]. Studies conducted in different parts of the world have demonstrated different levels of agreement between self-reported MC status and investigator assessed MC status. The result of this study show that there is a high level of agreement between self-assessment and investigator assessment in PNG and suggests self-reporting of MC status to be highly reliable among men in PNG.

## Supporting Information

S1 Table Regression analysis of demographic factors with accordance. (DOCX)
