# Supplementary material for: Men in Papua New Guinea Accurately Report Their Circumcision Status
Source: PLoS One. 2015 Apr 13;10(4):e0123429. doi: 10.1371/journal.pone.0123429 (PMC4395044; doi:10.1371/journal.pone.0123429)
Supplement: S1 Table — (DOCX) [file pone.0123429.s001.docx]

| Parameter | β | SE | OR | 95%CI | P |
| --- | --- | --- | --- | --- | --- |
| Age | -0.38 | 0.023 | 0.962 | 0.919 -1.008 | 0.102 |
| Education | -0.778 | 1.069 | 0.459 | 0.570 -3.732 | 0.467 |
| Employment status | 1.137 | 1.066 | 3.117 | 0.386 -25.204 | 0.286 |

S1 Table. Regression analysis of demographic factors with accordance
